# Taselisib, a PI3K Inhibitor, Alleviates Neuropathic Pain through HIF-1α-Mediated Angiogenesis in a Rat Model of Chronic Constriction Injury

**DOI:** 10.1159/nim/adxag005

**Published:** 2026-09-09

**Authors:** Hai-Ping You, Li-Hong Zhang, Chui-Yu Li, He-Fan He, Zhi-Yuan Chen, Hong-Geng Wang, Wei-Feng Liu, Chong-Jun Xu

**Affiliations:** Department of Anesthesiology, The Second Affiliated Hospital of Fujian Medical University, Quanzhou, China

**Keywords:** Neuropathic pain, Chronic constriction injury, HIF-1α, PI3K, Taselisib

## Abstract

**Introduction:**

Neuropathic pain is associated with hypoxia, inflammation, and metabolic dysregulation, in which hypoxia-inducible factor-1α (HIF-1α) plays a key regulatory role. This study investigates whether phosphoinositide 3-kinase (PI3K) inhibition by taselisib alleviates neuropathic pain via suppression of HIF-1α-mediated pathological signaling.

**Methods:**

In this study, a rat model of chronic constriction injury (CCI) was used to investigate whether neuropathic pain could be alleviated by taselisib, a selective PI3K inhibitor. Taselisib was administered daily, with or without co-administration of dimethyloxalylglycine (DMOG), a HIF-1α activator. Mechanical allodynia and thermal hyperalgesia were evaluated using behavioral tests. mRNA level in the dorsal root ganglia (DRG) was quantified by RT-qPCR, whereas protein levels were assessed by Western blotting, immunofluorescence, and metabolic assays were performed to assess angiogenesis, glycolytic activity, and inflammatory responses.

**Results:**

Taselisib significantly alleviated mechanical allodynia and thermal hyperalgesia in CCI rats, effects that were attenuated by co-administration of the HIF-1α activator DMOG. Taselisib suppressed CCI-induced upregulation of HIF-1α in DRG at both transcript and protein levels, and this suppression was reversed by DMOG. Downstream targets of HIF-1α, including mTOR, VEGFA, GLUT1, PFKM, and PDK1, were similarly downregulated by taselisib and partially restored upon HIF-1α activation. Metabolically, taselisib reduced lactate accumulation and restored ATP levels, indicating inhibition of glycolytic reprogramming. Taselisib also attenuated systemic and DRG-localized inflammatory responses, including IL-6, IL-1β, and NF-κB p65 expression; these anti-inflammatory effects were mitigated by DMOG.

**Conclusion:**

Taselisib alleviates neuropathic pain by disrupting HIF-1α-mediated angiogenic, inflammatory, and metabolic signaling. Targeting the PI3K-HIF-1α axis offers a promising therapeutic approach.

## Introduction

Neuropathic pain is a complex pathological condition associated with peripheral nerve injury, inflammation, and metabolic dysfunction. Increasing evidence suggests that pathological angiogenesis plays a critical role in the onset and maintenance of neuropathic pain [1, 2]. Particularly, aberrant neovascularization within the dorsal root ganglia (DRG) and injured peripheral nerves contributes to mechanical compression and pro-nociceptive neuronal sensitization [3]. Vascular endothelial cells further exacerbate this process by releasing pro-inflammatory cytokines, such as interleukin-6 (IL-6) and tumor necrosis factor-α (TNF-α), as well as neurotrophic factors, including nerve growth factor [4, 5]. These factors interact synergistically through neuroimmune and neurovascular pathways to potentiate nociceptive signal transmission [6]. This cascade is driven partially by dysregulated activation of the vascular endothelial growth factor (VEGF) signaling pathway [2], resulting in increased vascular permeability and the formation of a pro-inflammatory microenvironment [1]. These events culminate in heightened neuronal excitability and the persistent propagation of pain signals [7]. Notably, neurovascular unit dysfunction is not limited to the peripheral nervous system but also contributes to structural and functional remodeling within the central nervous system [8], offering novel insights into the pathophysiological mechanisms of neuropathic pain.

Hypoxia-inducible factor-1α (HIF-1α), a main transcriptional regulator of angiogenesis and glycolytic metabolism, is significantly upregulated under conditions of nerve compression and tissue hypoxia. It activates endothelial cells by inducing VEGFA expression, while engaging in cross talk with inflammatory pathways such as nuclear factor-κB (NF-κB) [9, 10]. In DRG neurons, HIF-1α promotes metabolic reprogramming through the upregulation of key glycolytic mediators, including glucose transporter 1 (GLUT1), phosphofructokinase muscle isoform, and pyruvate dehydrogenase kinase 1 (PDK1) [11, 12]. These alterations contribute to a self-reinforcing cycle of hypoxia, inflammation, and aberrant angiogenesis [1, 13]. Although HIF-1α is increasingly recognized as a central mediator in the pathophysiology of neuropathic pain [14, 15], pharmacological strategies targeting this molecule remain in the early stages of development [16]. The mechanistic basis of its involvement, particularly via PI3K/AKT/mTOR/VEGFA signaling [17], and its therapeutic potential [18] warrant further investigation.

In a previous work, miR-20b-5p was shown to attenuate chronic constriction injury (CCI)-induced neuropathic pain by targeting AKT3 and suppressing pro-inflammatory signaling [19]. More recently, taselisib, a selective inhibitor of phosphoinositide 3-kinase (PI3K), was reported to exert analgesic effects in CCI models through inhibition of the PI3K-AKT signaling cascade [20]. Building on these findings, the present study was designed to determine whether taselisib alleviates neuropathic pain via suppression of HIF-1α-mediated angiogenesis, inflammation, and glycolytic activation. In a rat model of CCI, pharmacological inhibition of PI3K by taselisib was found to reduce HIF-1α expression and the transcription of its downstream effectors. Notably, reactivation of HIF-1α was sufficient to reverse these analgesic effects, implicating HIF-1α as a critical downstream mediator of PI3K-dependent modulation of neuropathic pain.

## Materials and Methods

### Rat Model of Neuropathic Pain

Male Sprague Dawley rats (Vital River, Beijing, China), weighing 250–280 grams, were used. The study was approved by the Second Affiliated Hospital of Fujian Medical University. Neuropathic pain was induced using the CCI model. Under general anesthesia, the left sciatic nerve was exposed via a standardized surgical approach as previously described [19]. In the CCI group, four loose ligatures using 4-0 chromic gut sutures were placed around the nerve at approximately 1-mm intervals to induce partial nerve injury. In the sham group, the nerve was exposed without ligation.

### Drug Preparation

Taselisib (HY-13898) and dimethyloxalylglycine (DMOG; HY-15893) were obtained from MedChemExpress (Shanghai, China). Taselisib was suspended in a vehicle containing 0.5% methylcellulose and 0.2% Tween-80. DMOG, a cell-permeable, competitive inhibitor of prolyl hydroxylases (HIF-PHDs) that stabilizes and activates hypoxia-inducible factor-1α (HIF-1α), was dissolved in sterile saline.

### Drug Treatment

Rats were randomly assigned to four experimental groups: sham, CCI with vehicle treatment (CCI group), CCI with taselisib treatment (Tase group), and CCI with combined taselisib and DMOG treatment (Tase + DMOG group). Taselisib was administered orally at a dose of 10 mg per kg once daily. In the Tase + DMOG group, rats received taselisib as described, together with DMOG administered intraperitoneally at a dose of 60 mg per kg. Control animals received equivalent volumes of vehicle via the corresponding routes. Mechanical allodynia and thermal hyperalgesia were assessed on day 0 as baseline, and on postoperative days 3, 7, 14, and 21. After the final behavioral evaluation, animals were euthanized, and tissue samples were collected for molecular and biochemical analyses.

### Behavioral Assays

All behavioral tests were performed according to the IASP guidelines for animal pain research. Behavioral assessments were conducted between 8:00 a.m. and 2:00 p.m. by an independent investigator blinded to group allocation. Before testing, rats were acclimated to the testing room for 2 consecutive days, with at least 1 h of habituation each day.

Mechanical paw withdrawal threshold was assessed using an electronic von Frey anesthesiometer (Model 2390, IITC Life Science Inc., USA). Rats were placed individually in transparent Plexiglas chambers on a metal mesh floor and allowed to acclimate for 30 min before testing. The von Frey probe was applied perpendicularly to the mid-plantar surface of the left hind paw with gradually increasing force until a brisk paw withdrawal or paw licking response was observed. The withdrawal force was automatically recorded in grams. To avoid tissue injury, a cutoff force of 50 g was applied. Each rat was tested three times at each time point, with a 10-min interval between trials, and the average value was used for analysis.

Thermal pain latency was assessed using a hot-plate apparatus (Model 35,100, Ugo Basile, Italy) maintained at 52 ± 0.5°C. Rats were placed individually on the center of the heated plate, and the latency to paw licking, paw withdrawal/lifting, or jumping was recorded. A 30-s cutoff was used to prevent thermal injury. Each rat was tested three times at each time point with 15-min intervals, and the mean latency was used for analysis.

### RNA Extraction and RT-qPCR

Total RNA was extracted from the spinal dorsal horn using standard protocols, and cDNA was synthesized using the Hifair^®^ III 1st Strand cDNA Synthesis SuperMix (Yeasen, Shanghai, China). Gene expression analysis was performed by RT-qPCR using SYBR Green chemistry. GAPDH was used as the internal control. DRG tissue was used to assess mRNA expression of *Hif1a*, *Mtor*, *Vegfa*, *Glut1*, *Pfkm*, and *Pdk1* in *Rattus norvegicus*. The following primer sequences were used: *Hif1a* (F: 5′-CAC​AGC​AGA​CAC​AGA​CAG​AAG​CA-3′, R: 5′-TAT​CGA​GGC​TGT​GTC​GAC​TGA​A-3′), *Mtor* (F: 5′-GGA​GAT​GGC​TGT​GTT​CAA​GG-3′, R: 5′-CAG​GGT​CTT​CAC​AGC​CAT​CT-3′), *Vegfa* (F: 5′-TAC​CTC​CAC​CAT​GCC​AAG​TG-3′, R: 5′-TGG​AAG​ATG​TCC​ACC​AGG​GT-3′), *Glut1* (F: 5′-CGG​GCC​AAG​AGT​GTG​CTA​AA-3′, R: 5′-TGA​CGA​TAC​CGG​AGC​CAA​TG-3′), *Pfkm* (F: 5′-CAG​GGA​CAG​CAA​CAG​GTG​AT-3′, R: 5′-TGC​CTT​GTG​TTT​GGG​TTG​TT-3′), *Pdk1* (F: 5′-GCA​GCC​AGT​GGA​AGA​CAT​CA-3′, R: 5′-TCC​GCT​GTA​GCC​ATC​TTC​AC-3′), and *Gapdh* (F: 5′-TCA​AGA​AGG​TGG​TGA​AGC​AG-3′, R: 5′-AGG​TGG​AAG​AAT​GGG​AGT​TG-3′).

### Western Blot

DRG tissues were rapidly collected and homogenized in ice-cold RIPA lysis buffer (X-Blot, Suzhou, China) supplemented with protease and phosphatase inhibitors (Selleck, Shanghai, China). Tissue lysates were centrifuged at 12,000 *g* for 15 min at 4°C, and the supernatants were collected. Protein concentrations were determined using a BCA protein assay kit (Yeasen, Shanghai, China). Equal amounts of protein were separated by SDS-PAGE and transferred onto PVDF membranes (Millipore, MA, USA). Subsequently, the membranes were blocked with 5% non-fat milk or 5% BSA in TBST for 1 h at room temperature. For phosphorylated protein, it was blocked using 5% BSA. The membranes were then incubated overnight at 4°C with the following primary antibodies: anti-HIF-1α (#14179, Cell Signaling Technology [CST], MA, USA), anti-phospho-mTOR (Ser2448) (#2971, CST), anti-mTOR (#2972, CST), anti-NF-κB p65 (#8242, CST), VEGFA (ab214424, Abcam, Cambridge, UK), and anti-GAPDH (#5174, CST). After washing three times with TBST, the membranes were incubated with HRP-conjugated secondary antibody for 1 h at room temperature. Protein bands were visualized using an enhanced chemiluminescence detection system (X-Blot) and quantified using ImageJ software. GAPDH was used as the loading control.

### Lactic Acid and ATP Detection

Lactic acid (LA) and ATP levels in DRG tissues were measured using a lactate assay kit (Sigma-Aldrich, St. Louis, MO, USA; Cat. No. MAK064) and an ATP Detection Assay Kit-Luminescence (Cayman Chemical, Ann Arbor, MI, USA; Item No. 700410), respectively, according to the manufacturers’ instructions. For LA detection, absorbance was measured at 570 nm using a Synergy H1MD multimode microplate reader (BioTek, Winooski, VT, USA). For ATP detection, luminescence intensity was recorded using the luminometer mode of the same microplate reader.

### Enzyme-Linked Immunosorbent Assay

Serum and DRG tissue levels of IL-6 and IL-1β were measured using rat IL-6 (Cat. No. ERA32RB) and IL-1β (Cat. No. ERIL1B) enzyme-linked immunosorbent assay (ELISA) kits (Thermo Fisher Scientific, MA, USA), according to the manufacturer’s instructions. DRG tissues were homogenized in lysis buffer and centrifuged to collect the supernatants. Blood samples were centrifuged at 3,000 rpm for 15 min at 4°C to obtain serum. Absorbance was measured at 450 nm using a Synergy H1MD multimode microplate reader (BioTek, Winooski, VT, USA).

### Immunofluorescence

Rats were anesthetized using isoflurane and perfused transcardially with 0.9% saline, followed by 4% paraformaldehyde. The L4-L5 spinal cord segments were dissected after fixation in 4% paraformaldehyde at 4°C overnight, and cryosectioned into 20-μm sections. Tissue sections were blocked with 1% BSA for 2 h at room temperature, then incubated with recombinant anti-HIF-1α antibody (ab179483, Abcam, MA, USA) overnight at 4°C. After rinsing, sections were incubated with secondary antibody conjugated with Alexa Fluor 488 (Jackson ImmunoResearch, PA, USA) for 2 h at room temperature. A minimum of five sections from each animal were examined under a Leica SP8 Gated STED confocal microscope (Leica Microsystems, Wetzlar, Germany), and the average fluorescence intensity was calculated using ImageJ software after background correction.

### Statistical Analysis

Data are presented as mean ± standard deviation (SD). Group comparisons were performed using the Brown-Forsythe ANOVA, followed by Dunnett’s T3 multiple comparisons test to account for unequal variances. Statistical significance was determined when *p* values <0.05.

## Results

### HIF-1α Activator Attenuated the Effects of Taselisib on Mechanical Allodynia and Thermal Hyperalgesia in CCI Rats

To investigate whether HIF-1α modulates taselisib-induced analgesia, mechanical and thermal nociceptive responses were assessed using the Von Frey and hot plate assays, respectively, over 21 days following CCI. In the Von Frey test, CCI markedly reduced paw withdrawal thresholds compared to sham controls, indicating pronounced mechanical allodynia (Fig. 1a). Taselisib treatment significantly elevated withdrawal thresholds from day 7 onward compared to the vehicle group, reflecting a robust antiallodynic effect (Fig. 1a). However, co-treatment with DMOG attenuated this effect, as evidenced by significantly lower thresholds in the Tase + DMOG group compared to taselisib alone (Fig. 1a). Similarly, in the hot plate assay, CCI significantly reduced paw withdrawal latency, consistent with thermal hyperalgesia (Fig. 1b). Taselisib significantly prolonged withdrawal latency, whereas concurrent DMOG administration partially abolished this effect, with latencies significantly shorter than those observed in the taselisib group (Fig. 1b). These findings demonstrated that activation of HIF-1α compromised the analgesic efficacy of taselisib in both mechanical and thermal modalities.

**Fig. 1.**
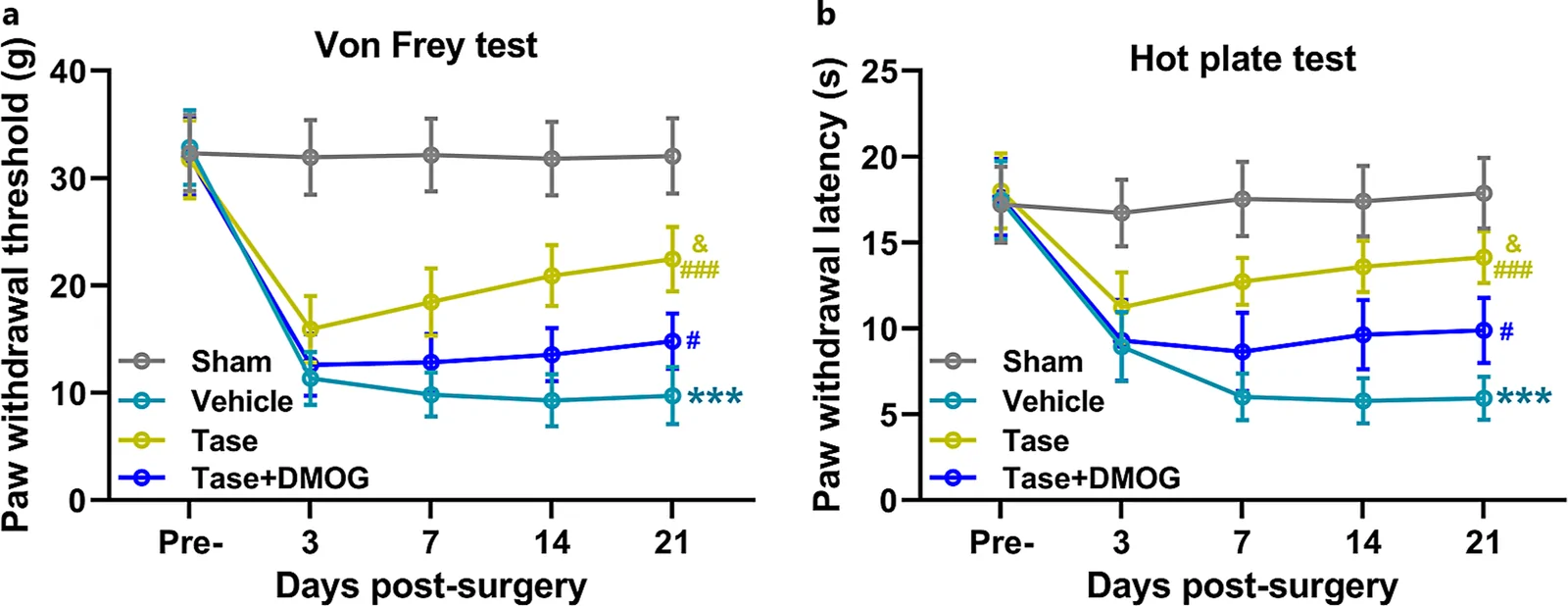
HIF-1α activator attenuated the effects of taselisib on mechanical allodynia (**a**) and thermal hyperalgesia (**b**) in CCI rats. *n* = 8 for each group. Data were presented as mean ± SD. ***p < 0.001 compared to Sham. #*p* < 0.05, ###*p* < 0.001 compared to CCI group. &*p* < 0.05 compared to the single taselisib-treated group. Brown-Forsythe ANOVA test followed by Dunnett’s T3 multiple comparisons test.

### Taselisib Inhibited HIF-1α Expression in the DRG of CCI Rats

To investigate whether PI3K inhibition alters HIF-1α expression in neuropathic pain, HIF-1α levels were assessed in the DRG. Immunofluorescence analysis revealed a marked upregulation of HIF-1α in the DRG of CCI rats compared to sham controls, while taselisib treatment significantly suppressed HIF-1α level (Fig. 2a, b). However, the suppression effect was reversed by the co-administration of DMOG, which significantly restored HIF-1α staining intensity compared to the taselisib group (Fig. 2a, b). Consistent with the immunostaining results, HIF-1α mRNA levels were significantly elevated in the vehicle-treated group and reduced following taselisib administration, while DMOG partially restored its expression (Fig. 2c). Western blot analysis further confirmed these findings. HIF-1α protein was significantly increased in the CCI group, reduced by taselisib treatment, and re-elevated upon DMOG co-treatment (Fig. 2d, e). Collectively, these findings demonstrate that taselisib downregulates CCI-induced HIF-1α expression at both the transcript and protein levels and that pharmacological activation of HIF-1α is sufficient to antagonize this suppressive effect.

**Fig. 2.**
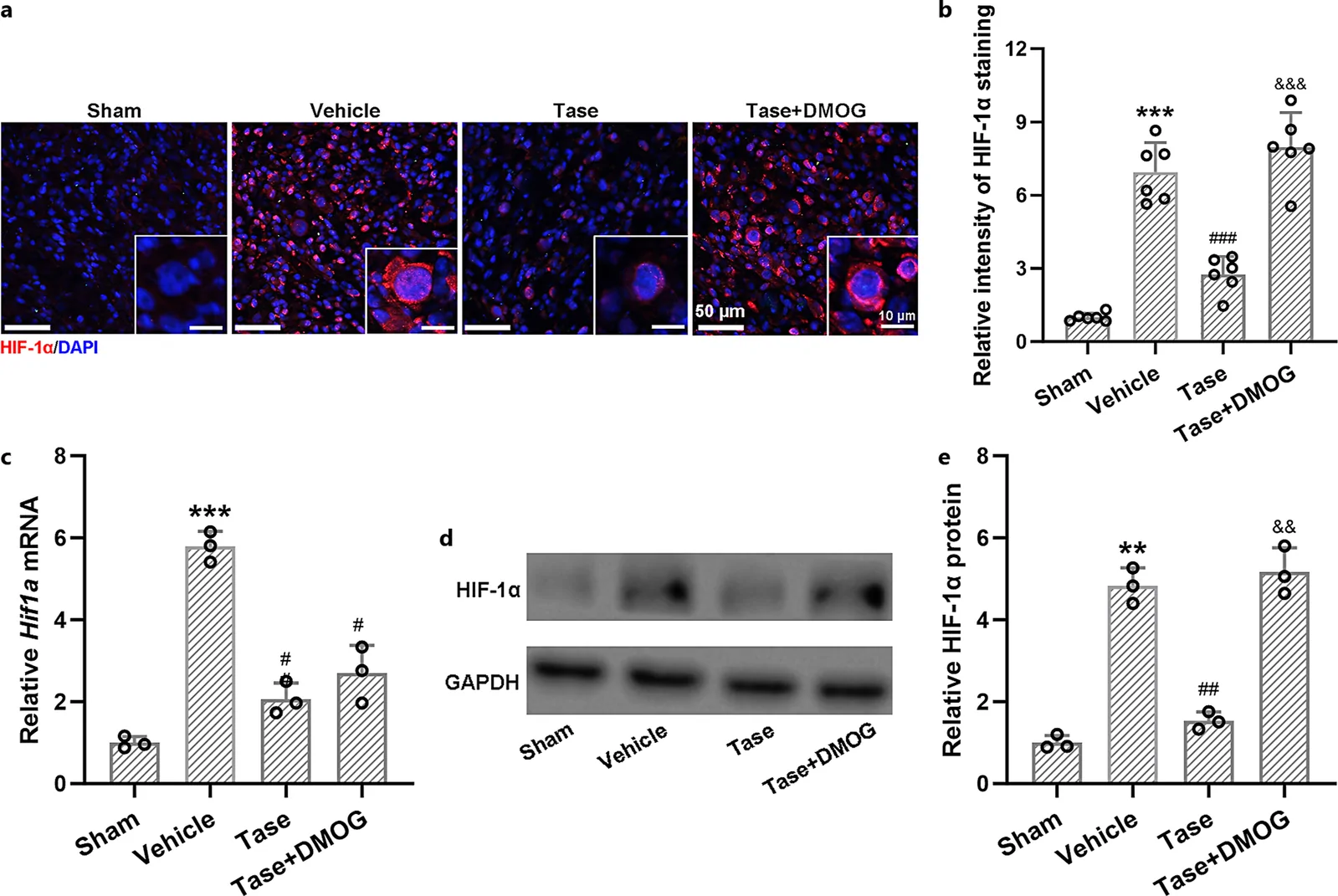
Taselisib inhibited HIF-1α expression in the DRG of CCI rats. **a** Representative immunofluorescence staining of HIF-1α in DRG of rats from different groups, and the relative fluorescence intensity was compared (**b**). *n* = 6 for each group. **c** The mRNA expressions of HIF-1α in DRG were measured by RT-qPCR. **d** Western blotting was used to measure the protein expression of HIF-1α in DRG of rats from different groups. GAPDH was used as a loading control, and the expressions were normalized to sham (**e**). *n* = 3 repeats for each group (6 tissue homogenates were mixed for each group). Data were presented as mean ± SD. **p < 0.01, ***p < 0.001 compared to Sham. #*p* < 0.05, ##*p* < 0.01, ###*p* < 0.001 compared to CCI group. &&*p* < 0.01, &&&*p* < 0.001 compared to single taselisib-treated group. Brown-Forsythe ANOVA test followed by Dunnett’s T3 multiple comparisons test.

### HIF-1α Activator Attenuated the Effects of Taselisib on the mTOR/VEGF Signal in DRG of CCI Rats

To explore the regulatory role of HIF-1α in taselisib-mediated suppression of the mTOR/VEGFA signaling pathway, the expression of mTOR and VEGFA was assessed at both transcript and protein levels in the DRG of CCI rats. RT-qPCR analysis revealed significant upregulation of *Mtor* and *Vegfa* mRNA in the vehicle-treated CCI group compared to sham controls, indicating activation of the mTOR/VEGFA axis in response to nerve injury (Fig. 3a, b). Taselisib treatment markedly reduced the expression of both genes, whereas co-treatment with DMOG partially restored their mRNA levels (Fig. 3a, b).

**Fig. 3.**
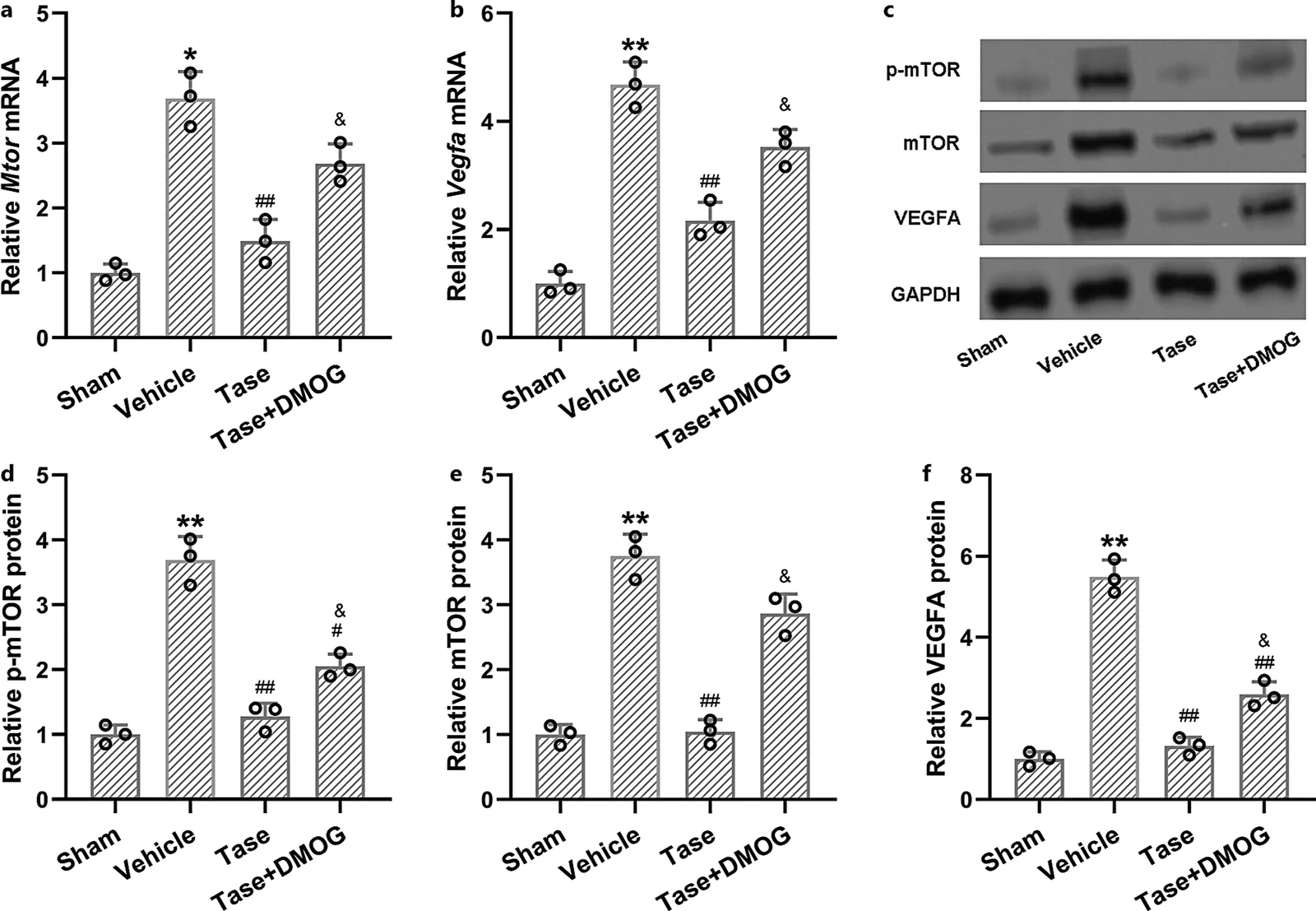
HIF-1α activator attenuated the effects of taselisib on the mTOR/VEGF signal in DRG of CCI rats. The mRNA expressions of mTOR (**a**) and VEGFA (**b**) in DRG were measured by RT-qPCR. **c** Western blotting was used to measure the protein expressions of p-mTOR, mTOR, and VEGFA in DRG of rats from different groups. GAPDH was used as a loading control, and the expressions were normalized to sham (**d–f**). *n* = 3 repeats for each group (6 tissue homogenates were mixed for each group). Data were presented as mean ± SD. *p < 0.05, **p < 0.01 compared to Sham. #*p* < 0.05, ##*p* < 0.01 compared to CCI group. &*p* < 0.05 compared to the single taselisib-treated group. Brown-Forsythe ANOVA test followed by Dunnett’s T3 multiple comparisons test.

Consistent with these transcriptional changes, Western blot analysis showed that protein levels of phosphorylated mTOR (p-mTOR), total mTOR, and VEGFA were all elevated in the vehicle group compared to sham (Fig. 3c–f) and were significantly downregulated by taselisib treatment (Fig. 3c–f). Importantly, co-administration of DMOG mitigated the suppressive effects of taselisib, as shown by increased expression of p-mTOR, mTOR, and VEGFA proteins compared to the taselisib group (Fig. 3c–f). These findings indicated that taselisib inhibited mTOR/VEGFA signaling in the DRG following CCI and that HIF-1α activation reversed this effect, suggesting that its analgesic action is at least partly mediated through the HIF-1α-mTOR-VEGF pathway.

### HIF-1α Activator Attenuated the Effects of Taselisib on Glycolytic Pathways in DRG of CCI Rats

To determine whether taselisib affects glycolytic reprogramming in neuropathic pain, lactate and ATP levels, as well as the expression of key glycolytic genes in the DRG of CCI rats were evaluated. CCI significantly increased LA and decreased ATP production in the DRG, compared to sham controls, indicating enhanced glycolytic flux and mitochondrial dysfunction (Fig. 4a, b). Taselisib treatment markedly reduced lactate levels and restored ATP content, suggesting an inhibitory effect on glycolysis and a partial recovery of energy homeostasis. However, co-administration of DMOG reversed these effects, as shown by elevated lactate and reduced ATP levels compared to taselisib monotherapy. Consistent with these metabolic changes, RT-qPCR analysis revealed that the mRNA expression of *Glut1*, *Pfkm*, and *Pdk1*, genes essential for glycolytic entry and regulation, was significantly upregulated in the vehicle group (Fig. 4c–e). Taselisib significantly suppressed the expression of all three genes, whereas co-treatment with DMOG partially restored the transcription (Fig. 4c–e). These findings suggest that taselisib suppresses CCI-induced glycolytic activation in the DRG, mediated by HIF-1α signaling.

**Fig. 4.**
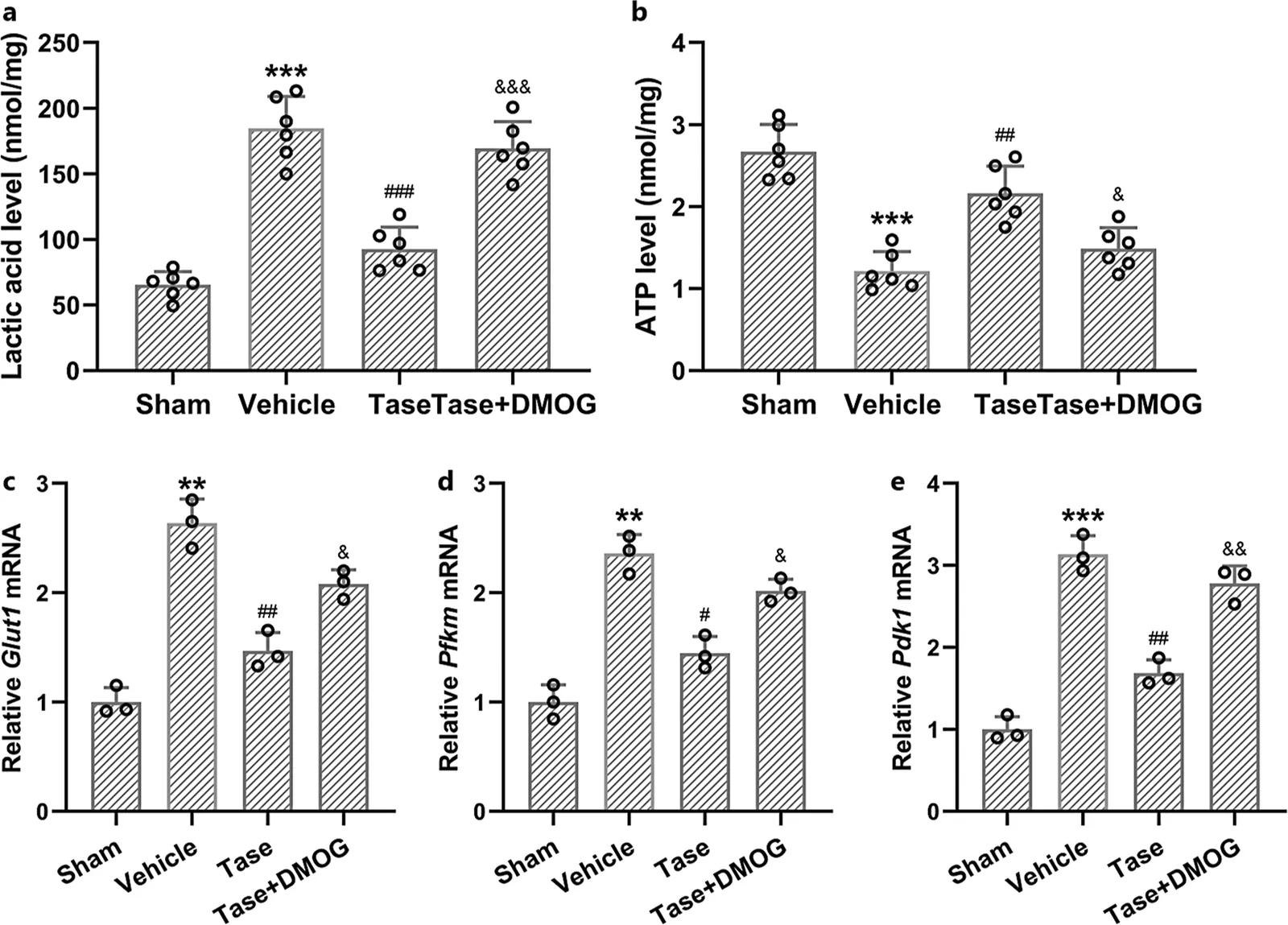
HIF-1α activator attenuated the effects of taselisib on glycolytic pathways in DRG of CCI rats. Lactic acid levels in DRG tissue (**a**) and ATP levels in DRG tissue (**b**) were compared. *n* = 6 for each group. The mRNA expressions of Glut1 (**c**), Pfkm (**d**), and Pdk1 (**e**) in DRG were measured by RT-qPCR. *n* = 3 repeats for each group (6 tissue homogenates were mixed for each group). Data were presented as mean ± SD. **p < 0.01, ***p < 0.001 compared to Sham. #*p* < 0.05, ##*p* < 0.01, ###*p* < 0.001 compared to CCI group. &*p* < 0.05, &&*p* < 0.01, &&&*p* < 0.001 compared to single taselisib-treated group. Brown-Forsythe ANOVA test followed by Dunnett’s T3 multiple comparisons test.

### HIF-1α Activator Attenuated the Effects of Taselisib on the Inflammatory Response in CCI Rats

To investigate whether the anti-inflammatory effects of taselisib are modulated by HIF-1α signaling, inflammatory markers were quantified in serum and DRG of CCI rats. In serum, CCI induced a marked increase in IL-6 and IL-1β concentrations, which was significantly attenuated by taselisib (Fig. 5a, b). The suppression by taselisib was partially reversed by co-administration of DMOG (Fig. 5a, b). In DRG tissues, taselisib similarly reduced CCI-induced elevations of IL-6 and IL-1β, an effect also blunted by DMOG treatment (Fig. 5c, d). At the molecular level, NF-κB p65 protein, a key transcriptional regulator of inflammatory signaling, was markedly upregulated in DRG tissues following CCI. Taselisib significantly suppressed NF-κB p65 expression, while DMOG partially reversed this effect (Fig. 5e, f). These findings indicate that taselisib attenuates CCI-induced inflammation in both peripheral and central compartments and that HIF-1α activation undermines this effect, via reactivation of NF-κB signaling.

**Fig. 5.**
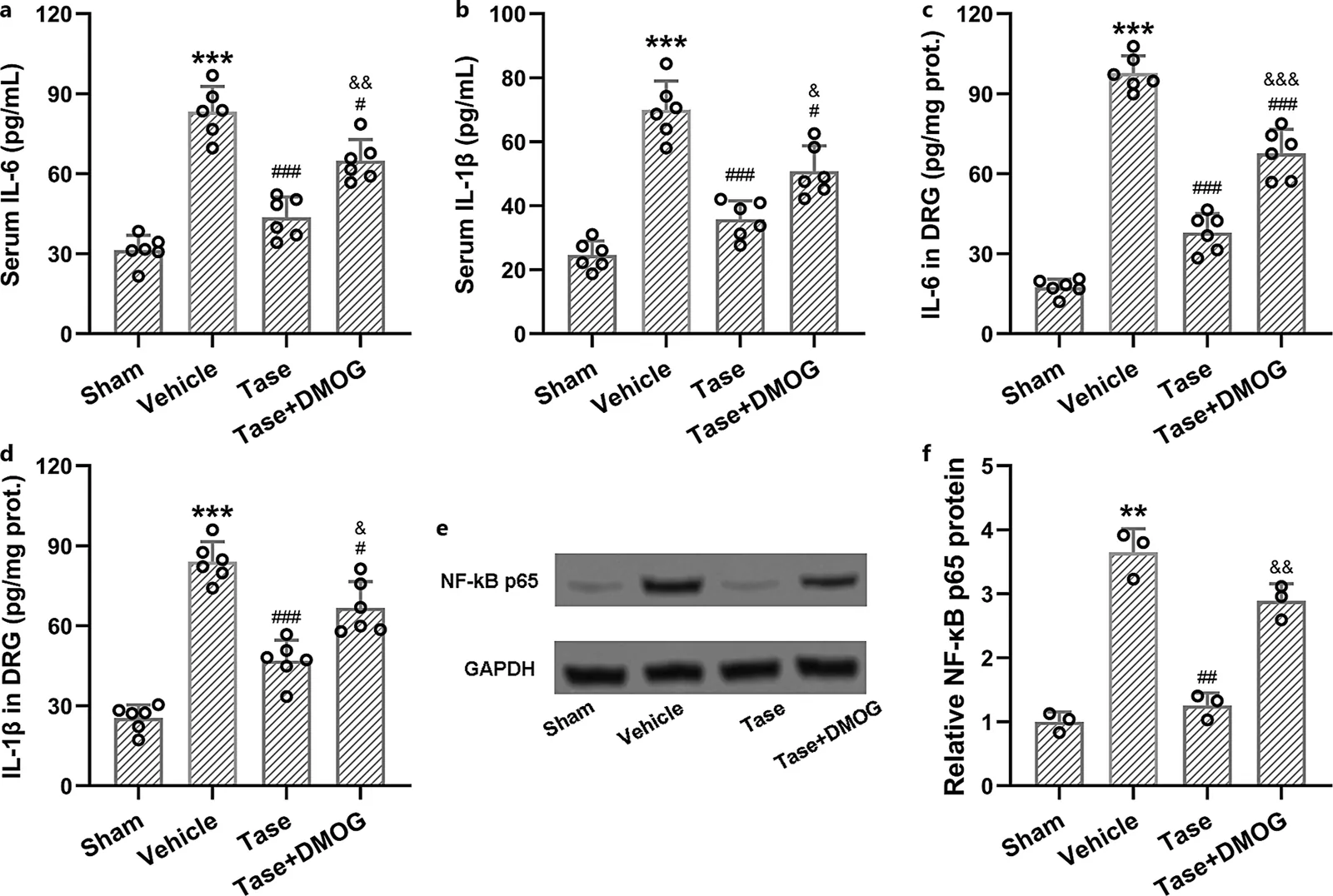
HIF-1α activator attenuated the effects of taselisib on the inflammatory response in CCI rats. The levels of serum IL-6 (**a**), serum IL-1β (**b**), IL-6 in DRG tissues (**c**), and IL-1β in DRG tissues (**d**) were measured by ELISA. *n* = 6 for each group. **e** Western blotting was used to measure the protein expression of NF-κB (p65) in DRG of rats from different groups. GAPDH was used as a loading control, and the expressions were normalized to sham (**f**). *n* = 3 repeats for each group (6 tissue homogenates were mixed for each group). Data were presented as mean ± SD. **p < 0.01, ***p < 0.001 compared to Sham. #*p* < 0.05, ##*p* < 0.01, ###*p* < 0.001 compared to CCI group. &*p* < 0.05, &&*p* < 0.01, &&&*p* < 0.001 compared to single taselisib-treated group. Brown-Forsythe ANOVA test followed by Dunnett’s T3 multiple comparisons test.

## Discussion

In this study, taselisib, a selective PI3K inhibitor, was shown to alleviate mechanical allodynia and thermal hyperalgesia in a rat model of CCI, an established model of neuropathic pain. Mechanistically, this analgesic effect was associated with the downregulation of HIF-1α and suppression of its downstream targets involved in angiogenesis, glycolysis, and inflammation within DRG. Notably, pharmacological activation of HIF-1α using DMOG was sufficient to reverse the antinociceptive effects of taselisib, restoring pain sensitivity and reactivating HIF-1α-dependent molecular pathways. These findings identify HIF-1α as a key downstream effector of PI3K signaling in neuropathic pain and suggest that its suppression may play a central role in the therapeutic effects of PI3K inhibition.

Our study identified a functional coupling between the PI3K-HIF-1α-mTOR/VEGF axis in the pathogenesis of neuropathic pain. PI3K signaling has been shown to stabilize HIF-1α expression through activation of the AKT/mTOR pathway [21], while HIF-1α transcriptionally upregulates both angiogenic factors, such as VEGF and glycolytic enzymes including *Glut1* and *HK2* [22], establishing a feed-forward regulatory loop. In the CCI model, this coupling was evident: taselisib suppressed PI3K activity and concomitantly downregulated the HIF-1α-mTOR/VEGF pathway, accompanied by reduced VEGFA expression and glycolysis-related molecular changes [23]. Although mTOR/VEGF signaling has previously been implicated in pain regulation [24], the current findings are the first to identify PI3K-HIF-1α as an upstream regulatory hub of this signaling cascade [25].

Although PI3K and HIF-1α have been individually implicated in pain modulation, the role of HIF-1α remains controversial, with evidence supporting both protective [26] and pro-nociceptive effects [27]. This study addresses the unresolved role of HIF-1α in neuropathic pain by providing multiple lines of mechanistic evidence. First, HIF-1α was identified as a downstream effector of PI3K signaling, as the antinociceptive effects of taselisib were reversed by pharmacological activation with DMOG [20]. Second, HIF-1α expression in the DRG was found to be dynamically regulated in parallel with pain-related behaviors in CCI rats, indicating spatiotemporal specificity. Third, extensive cross talk was observed between the PI3K-HIF-1α axis and downstream mTOR-VEGF and glycolytic pathways, revealing an integrated signaling network that has not been previously characterized, as previous studies have largely examined these pathways. Supporting this concept, Wen et al. [13] demonstrated that intrathecal administration of fumagillin alleviated neuropathic pain by inhibiting HIF-1α-mediated angiogenesis and glycolytic reprogramming in spinal neurons and astrocytes. While their work focused on central mechanisms, our findings extend the pathological relevance of HIF-1α to the peripheral nervous system. Specifically, inhibition of PI3K signaling was shown to suppress HIF-1α activity in DRG neurons, leading to coordinated downregulation of angiogenic, metabolic, and inflammatory pathways. Collectively, these results supported a model in which HIF-1α functioned as a conserved pro-nociceptive regulator across both central and peripheral components of the nociceptive system.

Taselisib demonstrated multimodal therapeutic potential by simultaneously targeting inflammation, VEGFA-related signaling, and metabolic dysregulation. It significantly attenuated neuroinflammatory responses by suppressing IL-6, IL-1β, and NF-κB, resembling the effects of conventional anti-inflammatory drugs. This finding is consistent with previous evidence that taselisib reduced pro-inflammatory cytokine production in CCI-induced neuropathic pain [20]. In parallel, it inhibited pathological angiogenesis via suppression of the HIF-1α-VEGF axis [28]. Notably, taselisib also reversed nerve injury-induced metabolic reprogramming by reducing lactate accumulation and restoring ATP levels [29]. The effects were dependent on HIF-1α-mediated regulation of glycolytic enzymes, including PFKM and PDK1. This multitarget profile may offer advantages over NSAIDs, which mainly target inflammation but often show limited efficacy and off-target effects in neuropathic pain.

Compared to conventional analgesics, PI3K inhibitors such as taselisib may provide distinct therapeutic advantages. First, they exhibit cell-type specificity by targeting PI3K-HIF-1α signaling in neurons and glial cells without broadly affecting systemic immunity [30]. Second, taselisib reverses a pain-associated Warburg-like glycolytic shift, a metabolic effect not addressed by traditional analgesics [25]. Third, a single dose leads to sustained suppression of HIF-1α and its downstream effectors, indicating prolonged efficacy [20]. Nonetheless, given the fundamental role of PI3K in metabolic regulation, careful optimization of dosing strategies and development of isoform-selective inhibitors will be crucial to minimize off-target effects [31].

The role of HIF-1α in pain appears to be context dependent; it may promote acute sensitization via PI3K/AKT activation [17], while its inhibition alone may be insufficient to reverse chronic pain circuits [27]. Our findings point to a therapeutic window in early-stage injury, where dual inhibition of PI3K and HIF-1α may yield synergistic effects. Moreover, selectively targeting HIF-1α in DRG or spinal cord tissue could reduce systemic side effects, such as impaired hypoxic responses. Thus, HIF-1α may function both as a mechanistic effector and a co-therapeutic target in precision pain management.

Available evidence suggests that the effects of DMOG are context dependent. As a prolyl hydroxylase inhibitor, DMOG appears to enhance HIF-1α signaling mainly under pathological conditions in which this pathway has already been activated. In a rat model of lumbar disk herniation-induced radiculitis, DMOG at 60 mg/kg reversed the analgesic effect of HIF-1α inhibition and restored the expression of glycolytic enzymes and inflammatory mediators in the DRG [12]. Similar pronociceptive effects of DMOG have been reported in thalamic hemorrhage-induced central post-stroke pain [32] and experimental autoimmune prostatitis, whereas DMOG alone did not induce pain-like behaviors or inflammatory gene expression in healthy mice [33]. Consistent with these findings, our pilot observations showed that DMOG treatment did not affect mechanical withdrawal thresholds or thermal pain latencies in sham-operated rats. Therefore, the reversal of taselisib-induced analgesia by DMOG in the present study is more possibly attributable to reactivation of HIF-1α-dependent pathways in the injured DRG, rather than to an independent pain-inducing effect of DMOG.

The difference between HIF-1α mRNA and protein levels after DMOG treatment is consistent with the known regulation of HIF-1α. HIF-1α is mainly regulated post-translationally. Under normoxia, HIF-1α protein is continuously synthesized but rapidly degraded through the ubiquitin-proteasome pathway [34]. Prolyl hydroxylation promotes HIF-α recognition by pVHL, followed by ubiquitination and proteasomal degradation [35, 36]. As a prolyl hydroxylase inhibitor, DMOG stabilizes HIF-1α protein rather than directly increasing Hif1α transcription [37]. This is also supported by evidence showing that PHD inhibition increases HIF-1α protein levels without a significant change in Hif1α mRNA expression [38]. Therefore, the increased protein level possibly reflects DMOG-induced stabilization of HIF-1α, which may reactivate downstream angiogenic, glycolytic, and inflammatory pathways.

Mechanistically, DMOG-induced HIF-1α activation may promote pain sensitization by enhancing glycolytic reprogramming through GLUT1, PFKM, and PDK1, leading to lactate accumulation and metabolic imbalance [12]. It may also promote M1 macrophage polarization and local inflammation [33], enhance mTOR/VEGFA-mediated angiogenic signaling [32], and amplify NF-κB- and NLRP3-related inflammatory responses, thereby contributing to a hypoxia-metabolism-inflammation axis involved in nociceptive sensitization [12, 32].

Taselisib may mediate analgesic effects by acting on multiple cell types. In DRG neurons, inhibition of the PI3K-HIF-1α axis may reduce glycolysis-related enzyme expression, attenuate metabolic reprogramming, and decrease neuronal excitability. Vascular endothelial cells may also be involved, as the HIF-1α/VEGFA pathway is closely associated with endothelial responses. Thus, reduced VEGFA expression after taselisib treatment may modulate VEGFA-related vascular signaling in the DRG, although direct effects on angiogenesis require further validation. In addition, immune and glial cells may also contribute to the observed effects, due to the role of HIF-1α in regulating pro-inflammatory mediators, such as IL-6 and IL-1β. However, the present study did not directly identify the cellular targets of taselisib. Cell type-specific HIF-1α conditional knockout models are needed to define the cell-specific roles of this pathway in neurons, endothelial cells, and immune/glial cells in future experiment.

These findings offer a framework for developing targeted therapies for neuropathic pain. HIF-1α, VEGF, and glycolytic byproducts such as lactate may serve as predictive biomarkers of treatment response [23]. Combining PI3K inhibitors with metabolic modulators could further improve efficacy. The development of isoform-selective PI3K inhibitors may also enhance tolerability by reducing off-target toxicity. Future studies in nonhuman primates are needed to assess the long-term safety and sustained analgesic effects of taselisib in clinically relevant models.

While our study provides compelling evidence that PI3K inhibition alleviates neuropathic pain through suppression of the HIF-1α-mTOR/VEGF and glycolytic pathways, several limitations should be acknowledged. First, only a single neuropathic pain model was employed, and the use of male rats limits the generalizability of the findings across sexes and pain etiologies. Second, pharmacological activation of HIF-1α using DMOG lacks spatial specificity, raising the possibility of off-target effects in nonneuronal tissues. Third, while downstream targets of HIF-1α, including VEGFA, GLUT1, and PFKM, were identified, the causal relationships among these mediators remain correlative. Future investigations using cell type-specific genetic approaches, such as targeted knockdown of HIF-1α in DRG neurons or vascular endothelial cells, will be necessary to define the precise cellular mechanisms underlying PI3K-HIF-1α signaling in neuropathic pain.

## Conclusion

Taselisib exerted potent antinociceptive effects in a rat model of neuropathic pain by selectively inhibiting the PI3K-HIF-1α signaling pathway. This intervention suppressed VEGFA-related signaling, glycolytic reprogramming, and neuroinflammation within the DRG. The reversal of these effects following pharmacological activation of HIF-1α supports its role as a key downstream effector of PI3K signaling. These findings identify the PI3K-HIF-1α axis as a central regulator of nociceptive plasticity and underscore its therapeutic potential in targeting the neurovascular-metabolic interface in neuropathic pain.

## Statement of Ethics

The study was approved by the Second Affiliated Hospital of Fujian Medical University and was performed in strict accordance with the NIH guidelines for the care and use of laboratory animals (NIH Publication No. 85-23 Rev. 1985).

## Conflict of Interest Statement

The authors declare they have no conflict of interest regarding this research study.

## Funding Sources

This work was supported by the 2025 Annual Joint Funding Project for Science and Technology Innovation in Fujian Province (#2025Y9418); Natural Science Foundation of Fujian Province (2024J01667); Quanzhou City Science and Technology Plan Project (#2024NY030); and Startup Fund for Scientific Research, Fujian Medical University (#2022QH1121). The funder had no role in the design, data collection, data analysis, and reporting of this study.

## Author Contributions

Hai-Ping You, Li-Hong Zhang, Chui-Yu Li, He-Fan He, Zhi-Yuan Chen, Hong-Geng Wang, Wei-Feng Liu, Chong-Jun Xu: data curation, analysis, validation, and manuscript preparation. Hai-Ping You and Chong-Jun Xu: supervision, project administration, funding acquisition, and writing-review and editing.

## Data Availability

The data that support the findings of this study are not publicly available due to privacy reasons but are available from Hai-Ping You upon request.

## References

[1] Sharma A , Behl T, Sharma L, Shah OP, Yadav S, Sachdeva M, . Exploring the molecular pathways and therapeutic implications of angiogenesis in neuropathic pain. Biomed Pharmacother. 2023;162:114693. 10.1016/j.biopha.2023.114693 37062217

[2] Stratton HJ , Boinon L, Gomez K, Martin L, Duran P, Ran D, . Targeting the vascular endothelial growth factor a/neuropilin 1 axis for relief of neuropathic pain. Pain. 2023;164(7):1473–88. 10.1097/j.pain.0000000000002850 PMC10277229 36729125

[3] Xie W , Luckemeyer DD, Qualls KA, Prudente AS, Berta T, Gu M, . Vascular motion in the dorsal root ganglion sensed by piezo2 in sensory neurons triggers episodic neuropathic pain. Neuron. 2025;113(11):1774–88.e5. 10.1016/j.neuron.2025.03.006 PMC12140901 40154477

[4] Yang F , Wang L, Song D, Zhang L, Wang X, Du D, . Signaling pathways and targeted therapy for rosacea. Front Immunol. 2024;15:1367994. 10.3389/fimmu.2024.1367994 PMC11439730 39351216

[5] Shen Y , Lin P. The role of cytokines in postherpetic neuralgia. J Integr Neurosci. 2025;24(4):25829. 10.31083/JIN25829 40302252

[6] Bararu-Bojan I , Badulescu OV, Badescu MC, Vladeanu MC, Plesoianu CE, Bojan A, . New insights into the pathophysiology of coronary artery aneurysms. Diagnostics. 2024;14(19):2167. 10.3390/diagnostics14192167 PMC11475291 39410571

[7] Ji H , Kim KR, Park JJ, Lee JY, Sim Y, Choi H, . Combination gene delivery reduces spinal cord pathology in rats with peripheral neuropathic pain. J Pain. 2023;24(12):2211–27. 10.1016/j.jpain.2023.07.007 37442406

[8] Jin S , Cho HJ. Cerebral hemodynamics as biomarkers for neuropathic pain in rats: a longitudinal study using a spinal nerve ligation model. Pain. 2025;166(1):171–82. 10.1097/j.pain.0000000000003332 38985168

[9] Bui BP , Nguyen PL, Lee K, Cho J. Hypoxia-inducible factor-1: a novel therapeutic target for the management of cancer, drug resistance, and cancer-related pain. Cancers. 2022;14(24):6054. 10.3390/cancers14246054 PMC9775408 36551540

[10] Gall T , Petho D, Erdelyi K, Egri V, Balla JG, Nagy A, . Heme: a link between hemorrhage and retinopathy of prematurity progression. Redox Biol. 2024;76:103316. 10.1016/j.redox.2024.103316 PMC11415884 39260060

[11] Li Y , Yin C, Jiang J, Yang H, Zhang F, Xing Y, . Tumor necrosis factor alpha-induced protein 8-like-2 controls microglia phenotype via metabolic reprogramming in bv2 microglial cells and responses to neuropathic pain. Int J Biochem Cell Biol. 2024;169:106541. 10.1016/j.biocel.2024.106541 38309648

[12] Ge H , Yan S, Yin M, Gao Y, Wang J, Wang Q, . Gua sha alleviates radiculitis-induced pain via hif-1alpha-mediated metabolic reprogramming pathway in rats. Pain Res Manag. 2025;2025:9923147. 10.1155/prm/9923147 PMC11932754 40130025

[13] Wen ZH , Wu ZS, Cheng HJ, Huang SY, Tang SH, Teng WN, . Intrathecal fumagillin alleviates chronic neuropathy-induced nociceptive sensitization and modulates spinal astrocyte-neuronal glycolytic and angiogenic proteins. Mol Neurobiol. 2025;62(1):246–63. 10.1007/s12035-024-04254-w 38837104

[14] Hua T , Kong E, Zhang H, Lu J, Huang K, Ding R, . Prmt6 deficiency or inhibition alleviates neuropathic pain by decreasing glycolysis and inflammation in microglia. Brain Behav Immun. 2024;118:101–14. 10.1016/j.bbi.2024.02.027 38402915

[15] Wu PZ , Yao J, Meng B, Qin YB, Cao S. Blood-nerve barrier enhances chronic postsurgical pain via the hif-1alpha/aquaporin-1 signaling axis. BMC Anesthesiol. 2023;23(1):381. 10.1186/s12871-023-02306-7 PMC10662690 37990154

[16] Yang DG , Gao YY, Yin ZQ, Wang XR, Meng XS, Zou TF, . Roxadustat alleviates nitroglycerin-induced migraine in mice by regulating hif-1alpha/nf-kappab/inflammation pathway. Acta Pharmacol Sin. 2023;44(2):308–20. 10.1038/s41401-022-00941-3 PMC9889379 35948752

[17] Yang L , Fu Q, Yang L, Zhang Y. Hif-1alpha/mmp-9 promotes spinal cord central sensitization in rats with bone cancer pain. Eur J Pharmacol. 2023;954:175858. 10.1016/j.ejphar.2023.175858 37356787

[18] Bellazzo A , Montico B, Guerrieri R, Colizzi F, Steffan A, Polesel J, . Unraveling the role of hypoxia-inducible factors in cutaneous melanoma: from mechanisms to therapeutic opportunities. Cell Commun Signal. 2025;23(1):177. 10.1186/s12964-025-02173-4 PMC11984274 40205422

[19] You H , Zhang L, Chen Z, Liu W, Wang H, He H. Mir-20b-5p relieves neuropathic pain by targeting akt3 in a chronic constriction injury rat model. Synapse. 2019;73(12):e22125. 10.1002/syn.22125 31381191

[20] You HP , Xu CJ, Zhang LH, Chen ZY, Liu WF, Wang HG, . Taselisib moderates neuropathic pain through pi3k/akt signaling pathway in a rat model of chronic constriction injury. Brain Res Bull. 2023;199:110671. 10.1016/j.brainresbull.2023.110671 37210013

[21] Zhong WJ , Liu T, Yang HH, Duan JX, Yang JT, Guan XX, . Trem-1 governs nlrp3 inflammasome activation of macrophages by firing up glycolysis in acute lung injury. Int J Biol Sci. 2023;19(1):242–57. 10.7150/ijbs.77304 PMC9760435 36594089

[22] Wang X , Liu Y, Hou H, Shao W, Huang D, Hao Z, . Mirna-29 aggravates myocardial infarction via inhibiting the pi3k/mtor/hif1alpha/vegf pathway. Aging. 2022;14(7):3129–42. 10.18632/aging.203997 PMC9037277 35378513

[23] Zhang Y , Jin H, Jia W, Liu Y, Wang Y, Xue S, . Ermiao san attenuating rheumatoid arthritis via pi3k/akt/mtor signaling activate hif-1alpha induced glycolysis. J Ethnopharmacol. 2025;345:119615. 10.1016/j.jep.2025.119615 40081512

[24] Xie X , Ma L, Xi K, Zhang W, Fan D. Microrna-183 suppresses neuropathic pain and expression of ampa receptors by targeting mtor/vegf signaling pathway. Cell Physiol Biochem. 2017;41(1):181–92. 10.1159/000455987 28214854

[25] Shan F , Zhang N, Yao X, Li Y, Wang Z, Zhang C, . Mechanosensitive channel of large conductance enhances the mechanical stretching-induced upregulation of glycolysis and oxidative metabolism in schwann cells. Cell Commun Signal. 2024;22(1):93. 10.1186/s12964-024-01497-x PMC10835878 38302971

[26] Lv B , Hua T, Li F, Han J, Fang J, Xu L, . Hypoxia-inducible factor 1 alpha protects mesenchymal stem cells against oxygen-glucose deprivation-induced injury via autophagy induction and pi3k/akt/mtor signaling pathway. Am J Transl Res. 2017;9(5):2492–9. PMC5446531 28559999

[27] Ludman T , Melemedjian OK. Bortezomib and metformin opposingly regulate the expression of hypoxia-inducible factor alpha and the consequent development of chemotherapy-induced painful peripheral neuropathy. Mol Pain. 2019;15:1744806919850043. 10.1177/1744806919850043 PMC6509977 31041875

[28] Peng W , Zhang S, Zhang Z, Xu P, Mao D, Huang S, . Jianpi jiedu decoction, a traditional Chinese medicine formula, inhibits tumorigenesis, metastasis, and angiogenesis through the mtor/hif-1alpha/vegf pathway. J Ethnopharmacol. 2018;224:140–8. 10.1016/j.jep.2018.05.039 29852266

[29] Kim D , Khin PP, Lim OK, Jun HS. Lpa/lpar1 signaling induces pgam1 expression via akt/mtor/hif-1alpha pathway and increases aerobic glycolysis, contributing to keratinocyte proliferation. Life Sci. 2022;311(Pt B):121201. 10.1016/j.lfs.2022.121201 36400203

[30] Wang X , Li X, Huang B, Ma S. Blocking mammalian target of rapamycin (mtor) improves neuropathic pain evoked by spinal cord injury. Transl Neurosci. 2016;7(1):50–5. 10.1515/tnsci-2016-0008 PMC5234518 28123821

[31] Bao YY , Zhong JT, Shen LF, Dai LB, Zhou SH, Fan J, . Effect of glut-1 and hif-1alpha double knockout by crispr/cas9 on radiosensitivity in laryngeal carcinoma via the pi3k/akt/mtor pathway. J Cell Mol Med. 2022;26(10):2881–94. 10.1111/jcmm.17303 PMC9907005 35415942

[32] Shi ZM , Jing JJ, Xue ZJ, Chen WJ, Tang YB, Chen DJ, . Stellate ganglion block ameliorated central post-stroke pain with comorbid anxiety and depression through inhibiting hif-1alpha/nlrp3 signaling following thalamic hemorrhagic stroke. J Neuroinflammation. 2023;20(1):82. 10.1186/s12974-023-02765-2 PMC10031944 36944982

[33] Meng T , Zhang Y, Wang H, Wu W, Peng W, Yue J, . Irf7 aggravates prostatitis by promoting hif-1alpha-mediated glycolysis to facilitate m1 polarization. Cell Mol Life Sci. 2025;82(1):90. 10.1007/s00018-025-05608-w PMC11846824 39985573

[34] Salceda S , Caro J. Hypoxia-inducible factor 1alpha (Hif-1alpha) protein is rapidly degraded by the ubiquitin-proteasome system under normoxic conditions. Its stabilization by hypoxia depends on redox-induced changes. J Biol Chem. 1997;272(36):22642–7. 10.1074/jbc.272.36.22642 9278421

[35] Jaakkola P , Mole DR, Tian YM, Wilson MI, Gielbert J, Gaskell SJ, . Targeting of hif-alpha to the von hippel-lindau ubiquitylation complex by o2-regulated prolyl hydroxylation. Science. 2001;292(5516):468–72. 10.1126/science.1059796 11292861

[36] Ivan M , Kondo K, Yang H, Kim W, Valiando J, Ohh M, . Hifalpha targeted for vhl-mediated destruction by proline hydroxylation: implications for o2 sensing. Science. 2001;292(5516):464–8. 10.1126/science.1059817 11292862

[37] Ogle ME , Gu X, Espinera AR, Wei L. Inhibition of prolyl hydroxylases by dimethyloxaloylglycine after stroke reduces ischemic brain injury and requires hypoxia inducible factor-1alpha. Neurobiol Dis. 2012;45(2):733–42. 10.1016/j.nbd.2011.10.020 PMC3286647 22061780

[38] Singh A , Wilson JW, Schofield CJ, Chen R. Hypoxia-inducible factor (hif) prolyl hydroxylase inhibitors induce autophagy and have a protective effect in an in-vitro ischaemia model. Sci Rep. 2020;10(1):1597. 10.1038/s41598-020-58482-w PMC6994562 32005890

